# The Evolution of NLR Inflammasome and Its Mediated Pyroptosis in Metazoa

**DOI:** 10.3390/ijms252011167

**Published:** 2024-10-17

**Authors:** Jiejie Sun, Jinyuan Leng, Linsheng Song

**Affiliations:** 1 Liaoning Key Laboratory of Marine Animal Immunology, Dalian Ocean University, Dalian 116023, China; lengjinyuan2023@163.com; 2 Liaoning Key Laboratory of Marine Animal Immunology & Disease Control, Dalian Ocean University, Dalian 116023, China; 3 Southern Laboratory of Ocean Science and Engineering (Guangdong, Zhuhai), Zhuhai 519000, China

**Keywords:** NLR, inflammasome, pyroptosis, metazoan, evolution

## Abstract

Nucleotide-binding oligomerization domain (NOD)-like receptor (NLR) inflammasomes are multiprotein signaling platforms that control the inflammatory response and coordinate antimicrobial defense. In the present study, the distribution of NLR, Caspase-1, and gasdermin (GSDM) homologues and their structural characteristics and evolutionary relationships were systematically analyzed in metazoa according to the genomes of species. In invertebrates, there were only NLRC and/or NLRD presented from sponge to amphioxus, and according to the evolutionary tree, NLR from sponge located in the most primitive position. Caspase-1 existed in some metazoan phyla (Brachiopoda, Ectoprocta, Arthropoda, Mollusca, Annelia, Nematoda, Platyelminthes, Coelenterate, and Porifera) and its activation sites were relatively conserved. The amino acid sequences and three-dimensional structures of N-terminal CARD/Death domain of NLR and Caspase-1 were similar in species from sponge to human. NLR and Caspase-1 co-existed in species of Brachiopoda, Mollusca, Annelia, Coelenterate, and Porifera. There was only GSDME or PJVK found in some phyla of invertebrates and their cleavage sites were conserved (DxxD). And it was predicted that the NLR inflammasome in inducing pyroptosis could occur in species of Brachiopoda, Mollusca, Annelia, and Coelenterate. These studies indicated that NLR inflammasome emerged early in sponges of metazoa, and NLR inflammasome in inducing pyroptosis first appeared in Coelenterate, suggesting that inflammasome and its mediated pyroptosis had existed in the early stage of metazoa, but they had been lost in many species during evolution.

## 1. Introduction

Throughout metazoan, inflammasomes are cytosolic multiprotein complexes that assemble in response to exogenous microbial invasions and damage signals [1,2,3]. The core sensor protein of inflammasome comes from the nucleotide-binding oligomerization domain (NOD)-like receptor (NLR) family. NLR is activated and oligomerized upon sensing microbial effectors or damage signals to recruiting the adaptor protein apoptosis-associated speck-like protein containing a caspase recruitment domain (CARD) (ASC). The activation of NLR inflammasome is critical for host defense because it facilitates the pro-IL-1β and pro-IL-18 into their mature forms [3] and initiates the lytic form of cell death, pyroptosis [4,5].

NLR inflammasomes as cytosolic protein complexes can sense various exogenous microbial invasions and damage signals [6]. NLR is widely characterized in vertebrates and its domain structure and function are best understood in these species [7,8,9]. NLR typically comprises a varied N-terminal effector domain, such as CARD or pyrin domain (PYD); a central nucleotide-binding oligomerization (NACHT) domain; and C-terminal leucine-rich repeats (LRRs) [10]. NLR oligomerizes into multiprotein complexes termed as inflammasomes when activated [11]. There are four subfamilies of NLRs, including NLRA, NLRB, NLRC, and NLRP [10]. In teleost, NLRP and NLRC homologues have been identified and their activation mechanisms are also preliminarily clarified [12,13]. Recently, NLRs have also been identified in basal metazoa and, surprisingly, have undergone a large expansion in some organisms, such as *Strongylocentrotus purpuratus*, *Nematosetella vectensis*, *Hydra magnipapilatta*, and *Amphimedon queenslandica* [7,8,14]; it remains unclear about the potential activation mechanism of NLR inflammasomes in species of lower metazoan phyla.

The current accepted paradigm is that there are individual NLR inflammasomes consisting of NLR, ASC, and Caspase-1 [1,15]. NLR upon sensing microbial effectors and danger signals recruits the adaptor protein ASC, which consists of a PYD and a CARD, through homotypic PYD-PYD (NLRP3) or CARD-CARD interaction (NLRP1 and NLRC4). The CARD of ASC is necessary to recruit Caspase-1 into the inflammasome complex, although CARD-containing receptors (NLRP1 and NLRC4) may also directly recruit Caspase-1 [1,16]. In teleost, an ASC homologue was also identified [12] and NLRP3 can interact with ASC to activate Caspase-B or directly bind with Caspase-B [17]; the ASC homologues or PYD-containing proteins are absent in the genomes of other even lower metazoa [18].

The activation of NLR inflammasomes leads to the cleavage of pro-IL-1β and pro-IL-18 into their mature forms IL-1β and IL-18, and also activates the proteolytic cleavage of gasdermin D (GSDMD) to induce pyroptosis [1]. To date, six members (GSDMA to GSDME and PJVK) are found in mammals [19]. The activation of NLRP3 and NLRC4 inflammasomes in human and mouse mediates GSDMD cleavage to generate an N-terminal fragment to induce pyroptosis. While in teleost, there only existed two GSDME homologues and the activated Caspase-1 could cleave pro-IL-1β into IL-1β, and also recognize and cleave GSDMEb to release its N-terminal domain to trigger pyroptosis [12,20]. The zebrafish NLRP3 inflammasome was demonstrated to mediate the cleavages of pro-IL-1β and GSDME [17]. Recently, in invertebrates, GSDME and pyroptosis were also found in oyster, coral, and hydra [21,22,23,24,25]; the potential activation mechanism of NLR inflammasome in GSDME-dependent pyroptosis in whole metazoan phyla is poorly understood.

Inflammasome has been well understood in vertebrates, and it plays crucial roles in host defense against pathogens [1,2,12]. However, in invertebrates, the studies on inflammasome structural composition, possible activation mechanisms, and mediation for pyroptosis remain largely unknown. In the present study, NLR, Caspase-1, and GSDM in basal metazoa from sponge to amphioxus were systematically analyzed with the objects to screen the distribution of NLR, Caspase-1, and GSDM in species of invertebrates. We also analyzed the composition and activation mechanism of NLR inflammasomes in these species and confirmed the existence of species with potential NLR inflammasome-mediated pyroptosis, providing insights into the evolution of inflammasomes and their mediated pyroptosis in metazoa.

## 2. Results

### 2.1. The Existence of NLRs in Metazoa and Their Structural Domain Composition

**Figure 1:**
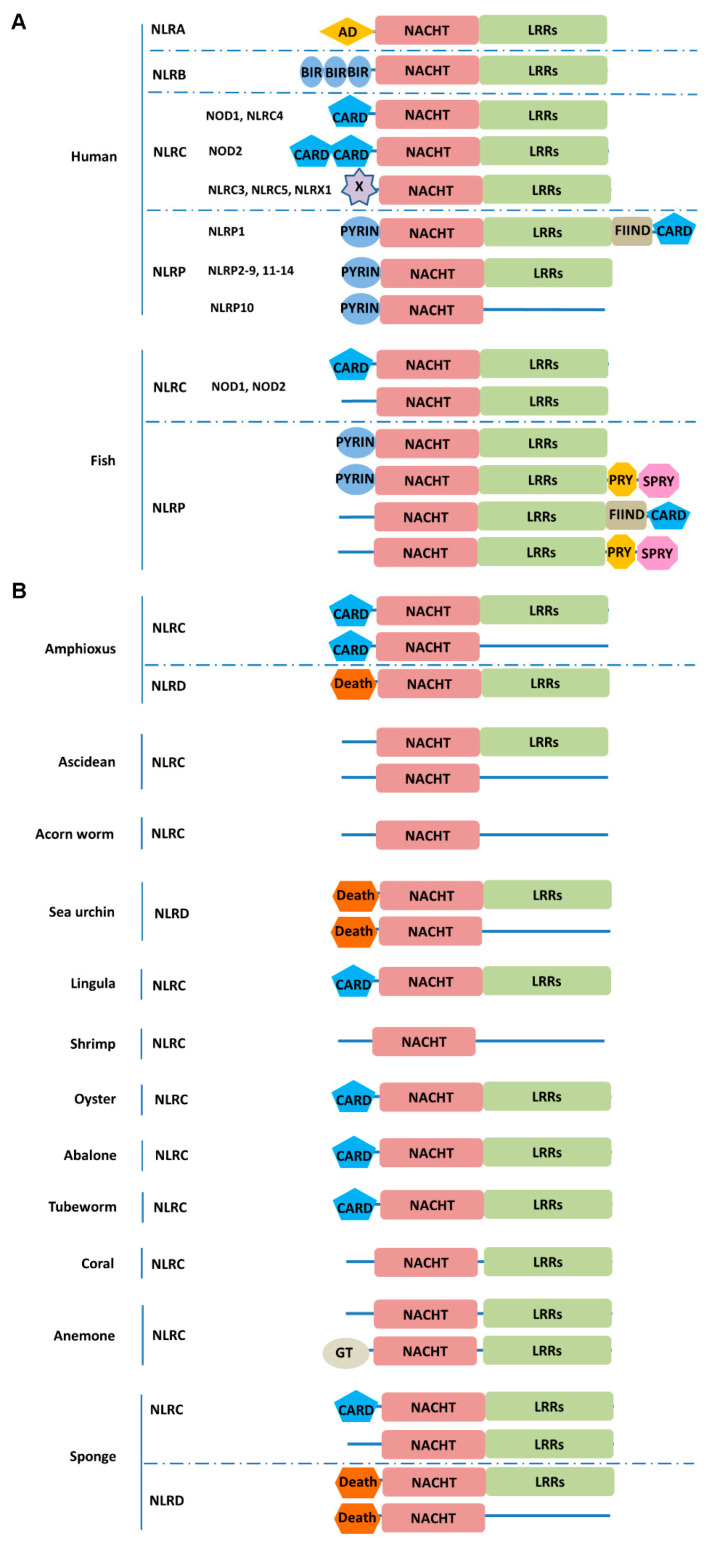
The different types and structural domains of NLRs in different metazoan phyla from sponge to human. (**A**) The different types and structural domains of NLRs in vertebrate phyla. (**B**) The different types and structural domains of NLRs in invertebrate phyla. The structural domains of NLRs were predicted by using SMART. AD: acidic transactivating; BIR (baculovirus IAP repeat), CARD (caspase-associated recruitment domain); LRR (leucine-rich repeat); NACHT, nucleotide-binding oligomerization; FIIND, domain with function to find; GT: glycosyltransferase; PRY-SPRY: B30.2 (Table S1).

In humans, there were four types of NLRs, including NLRA, NLRB, NLRC, and NLRP. They normally contained a NACHT domain and LRR repeats (LRRs) (Figure 1A). Furthermore, NLRA had an acidic transactivation (AD) domain, NLRB had three baculoviral IAP repeat (BIR) domains, NLRC had one or two CARD or unknown domain, and NLRP had a PYD. NLRP1 also contained function to find domain (FIIND)-CARD in the C-terminal domain. In fish, there were two types, NLRC and NLRP. NLRC had a N-terminal CARD or unknown domain, a NACHT domain, and LRRs. NLRP had a N-terminal PYD or unknown domain, a NACHT domain, and LRRs. In the C-terminal, some NLRPs also contained B30.2 (PRY-SPRY) domain or FIIND-CARD (Figure 1A). In amphioxus, there were two types of NLR. One was NLRC, which contained CARD-NACHT-LRRs or CARD-NACHT domain. The other was NLRD with Death-NACHT-LRRs domain. NLRD was also found in sea urchin and sponge, which contained Death-NACHT or Death-NACHT-LRRs domain. NLRC existed in most species of basal metazoa. Among which, in ascidian, acorn worm, shrimp, coral and anemone, NLRC lacked CARD, only containing NACHT-LRRs or NACHT domain. In lingula, oyster, abalone, tubeworm, and sponge, NLRC had the classical CARD-NACHT-LRRs domain like mammalian NLRC (Figure 1B).

In Craniata, the three-dimensional structure of fish *Dr*NLRC (XP_009297031.1) was similar with that of human *Hs*NLRC4, and that of fish *Dr*NLRC (XP_021325692.1) was similar with that of human *Hs*NLRC3. In invertebrates, the most NLRs from different species were more similar with that of *Hs*NLRC4, such as *Bl*NLRC (CAM1243784.1) and *Bl*NLRD (CAH1243793.1) of Cephalochordata, *Sp*NLRD (XP_030847555.1; XP_030850917.1) of Echinodermata, *La*NLRC (XP_013410021.1; XP_013419001.1), *Cg*NLRC (XP_011450878.1) of Mollusca, and *Pv*NLRC (XP_058959243.1) of Coelenterate. The three-dimensional structures of Death domain from *Bl*NLRD of Cephalochordata and *Sp*NLRD of Echinodermata were similar with that of CARD from other NLRCs (Figure S1).

### 2.2. The Evolutionary Relationship of NLRs in Metazoa

**Figure 2:**
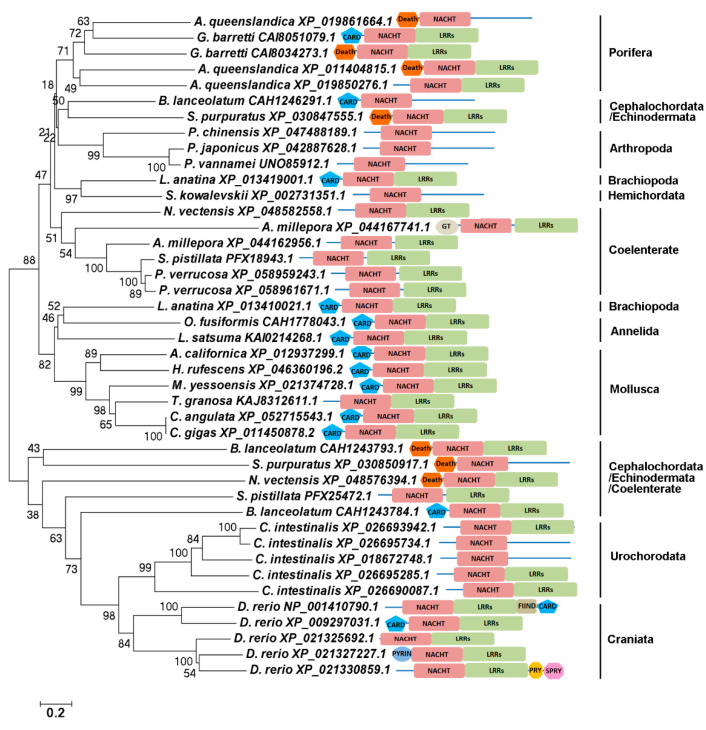
The evolutionary analysis of NLRs in different metazoan phyla from sponge to human. The phylogenetic tree was constructed with MEGA 7 software via the NJ method. The bootstrap of 1000 replications was conducted to evaluate the phylogenetic tree (Table S1).

The amino acid sequences of NLRs across metazoa were selected for constructing the evolutionary tree. In the evolutionary tree, there were two clearly branches and NLRs from the same phyla were mostly clustered together. All NLRs from *Danio rerio*, *Ciona intestinalis*, and some NLRs from *Branchiostoma lanceolatum*, *S. purpuratus*, and *Nematostella vectensis* were clustered into one branch. In another branch, NLRs from Porifera were the most evolutionarily primitive. NLRs from Brachiopoda, Mollusca, and Annelida were dropped into the sub-branch and they were relatively higher in evolution. NLRs from Porifera and Coelenterate had a distant evolutionary relationship. And those from Cephalochordata, Hemichordata, Echinodermata, Arthropoda, and Branchiopoda had a closer evolutionary relationship (Figure 2), suggesting that the evolutionary relationship of NLRs was different from that of species.

### 2.3. The Existence of ASCs and PYD in Metazoa

There were ASCs in human, mouse, and fish, and their amino acid sequences were conserved (Figure S2A). ASCs from human, mouse, and fish all contained a PYD and a CRAD. The three-dimensional structures of them were also similar. And the three-dimensional structure of PYRIN domain was similar to that of the CRAD domain (Figure S2B). There were no ASC or PYD in animal phyla except for Craniata (Figure S2C).

### 2.4. The Evolutionary Relationship of CARD-Containing Caspases in Metazoa

**Figure 3:**
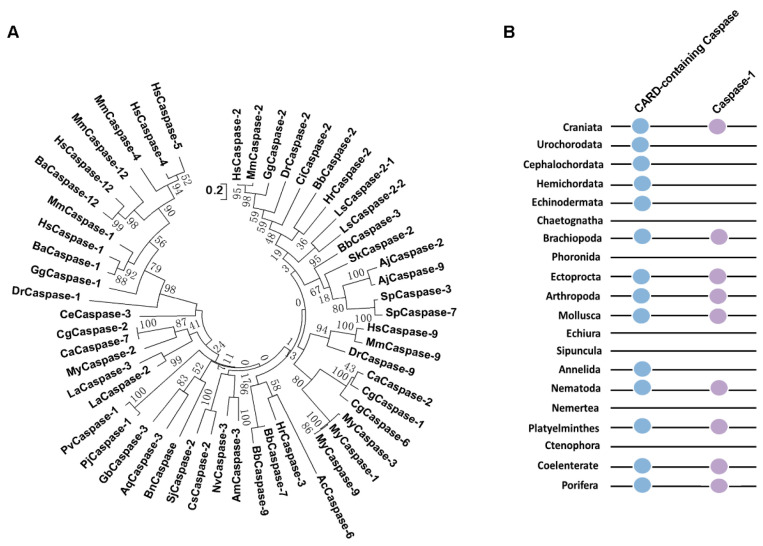
The evolutionary analysis of CARD-containing Caspases and their existence in different metazoan phyla. (**A**) The evolutionary analysis of CARD-containing Caspases in different metazoan phyla with MEGA 7 software. The following gene names were from those of specie genomes in NCBI database. *Geodia barretti* Caspase-3, CAI8040687.1; *A. queenslandica* Caspase-3-like, XP_003383519.1; *Acropora millepora* Caspase-3-like, XP_044168435.1; *Nematostella vectensis* Caspase-3, XP_032238419.2; *Schistosoma japonicum* Caspase-2, TNN06772.1; *Clonorchis sinensis* Caspase-2, KAG5446995.1; *Caenorhabditis elegans* Cell death protein 3 subunit p17, NP_001255708.1; *Lamellibrachia satsuma* Caspase-2-1, KAI0221544.1; *L. satsuma* Caspase-2-2, KAI0222024.1; *Crassostrea gigas* Caspase-1, XP_034337082.1; *C. gigas* Caspase-2-like, XP_011449817.2; *C. gigas* Caspase-6, XP_011423157.2; *C. angulata* Caspase-2-like, XP_052713160.1; *C. angulata* Caspase-7-like, XP_052717437.1; *Mizuhopecten yessoensis* Caspase-1-like, XP_021346856.1; *M. yessoensis* Caspase-2-like, XP_021351320.1; *M. yessoensis* Caspase-9-like, XP_021346855.1; *M. yessoensis* Caspase-3, OWF53614.1; *Haliotis rufescens* Caspase-2-like, XP_046326151.2; *H. rufescens* Caspase-3-like, XP_048247614.1; *Aplysia californica* Caspase-6, XP_012940622.1; *Lingula anatina* Caspase-2, XP_013414814.1; *L. anatina* Caspase-3, XP_013416922.1; *L. anatina* Caspase-9, XP_013407791.1; *Saccoglossus kowalevskii* Caspase-2-like, XP_006811879.1; *Apostichopus japonicus* Caspase-2, AOR82888.1; *A. japonicus* Caspase-9, PIK40152.1; *Strongylocentrotus purpuratus* Caspase-7, XP_030842855.1; *S. purpuratus* Caspase-3-like, XP_030843307.1; *Penaeus vannamei* Caspase-1-like, XP_027232060.1; *P. japonicus* Caspase-1-like, XP_042874475.1; *Branchiostoma belcheri* Caspase-2-like, XP_019642903.1; *B. belcheri* Caspase-3-like, XP_019644208.1; *B. belcheri* Caspase-9, KAI8516722.1; *B. belcheri* Caspase-7-like, XP_019623612.1; *Ciona intestinalis* Caspase-2-like, XP_002122917.1; *Danio rerio* Caspase-1, AWP39886.1; *D. rerio* Caspase-2, NP_001036160.1; *D. rerio* Caspase-9, AWP39896.1; *Homo sapiens* Caspase-1, NP_001214.1; *H. sapiens* Caspase-2, KAI4016188.1; *H. sapiens* Caspase-4, KAI4073885.1; *H. sapiens* Caspase-5, KAI2562572.1; *H. sapiens* Caspase-9, KAI4078765.1; *H. sapiens* Caspase-12, NP_001177945.2; *Mus musculus* Caspase-1, NP_033937.2; *M. musculus* Caspase-2, EDL13489.1; *M. musculus* Caspase-4, NP_001366249.1; *M. musculus* Caspase-9, AAH56447.1; *M. musculus* Caspase-12, AAH28979.1; *Gallus gallus* Caspase-1, XP_040506552.1; *G. gallus* Caspase-2, NP_001161173.2; *Balaenoptera acutorostrata* Caspase-1, XP_007191299.1; *B. acutorostrata* Caspase-12, XP_007191293.2. (**B**) The existence of CARD-containing Caspases and Caspase-1s in different metazoan phyla.

**Figure 4:**
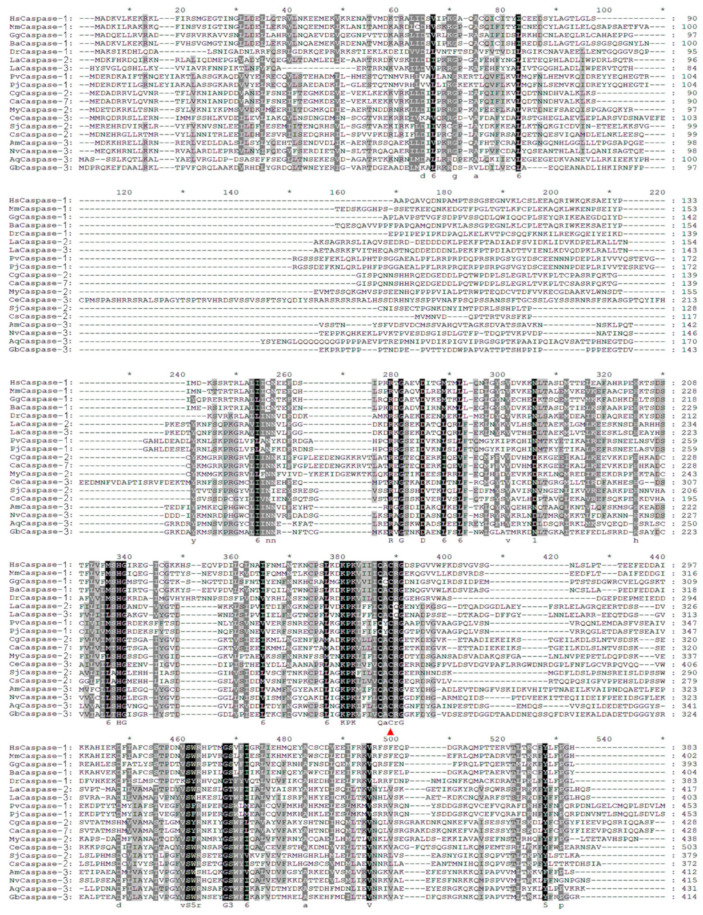
The multiple sequence alignment of Caspase-1s from different species of metazoan phyla. The multiple sequence alignment was generated via MEGA 7 and GeneDoc-32-v320 software. The red triangle indicated the activation sites.

CARD-containing Caspases from species of metazoa were used for screening Caspase-1 by using the evolutionary relationship. According to the evolutionary tree, there was no Caspase-4/5/12 homologue in fish and even lower metazoa (Figure 3A). Except for Caspase-4/5/12, the other Caspases from species of different metazoan phyla could be clearly divided into Caspase-1, Caspase-2, Caspase-9, and Caspase-11. CARD-containing Caspases were found in most metazoan phyla, and according to the evolutionary tree, though Caspase gene names annotated in genomes of species were different, there were Caspase-1 homologues in Craniata, Brachiopoda, Ectoprocta, Arthropoda, Mollusca, Annelida, Nematoda, Platyelminthes, Coelenterate, and Porifera (Figure 3A,B). The amino acid sequences of Caspase-1 homologues from species of different metazoan phyla were not conserved, but their activation sites (QACRG) were conserved (Figure 4).

### 2.5. The Sequence Alignment and Three-Dimensional Structure of the CARD/PYD/Death Domain from NLRs and Caspase-1s

**Figure 5:**
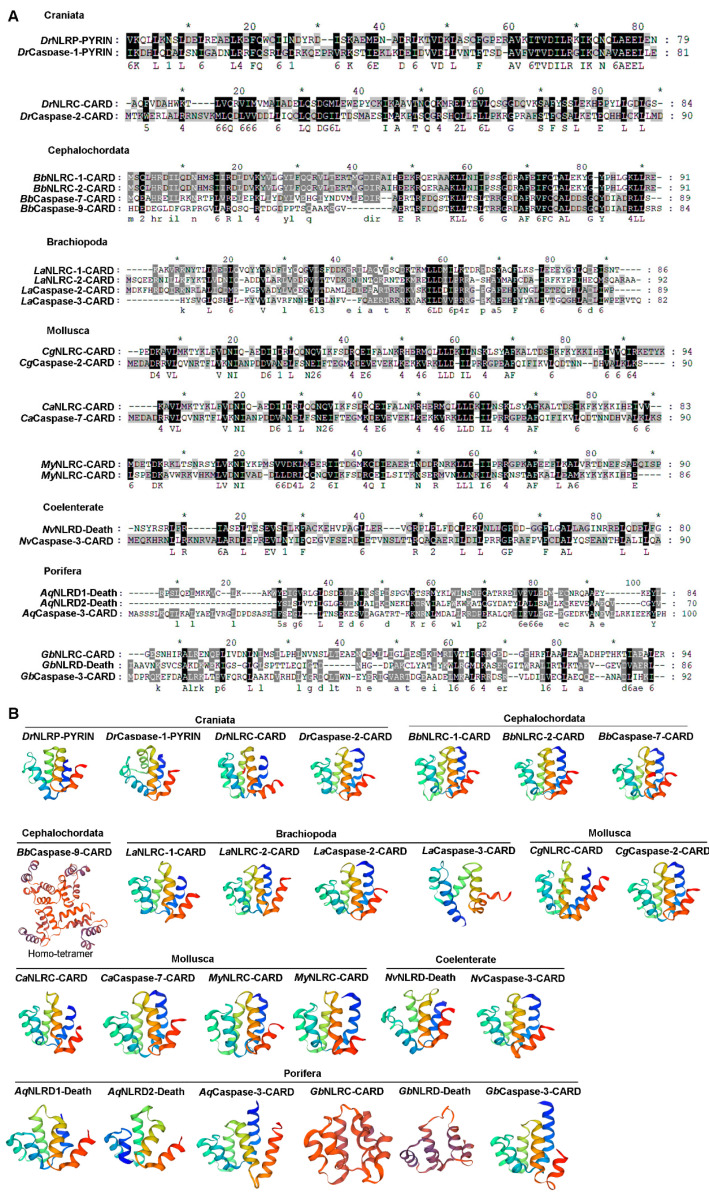
The multiple sequence alignment and three-dimensional structure of CARD/Death of NLRs and Caspase-1s from different species of metazoan phyla. (**A**) The multiple sequence alignment of PYD/CARD/Death domain in NLRs and Caspase-1s. (**B**) The three-dimensional structure of CARD/Death of NLRs and Caspase-1s.

The CARD/PYD/Death domain of NLRs and Caspase-1s from the same species were selected for analyzing the sequence similarity. They all had higher sequence similarity (Figure 5A), and their three-dimensional structures were also similar. Among which, CARD of *Bb*Caspase-9 from Cephalochordata and *Ls*NLRC from Annelida formed homotetramer and homodimer (Figure 5B).

### 2.6. The Sequence Alignment, Evolutionary Relationship, and Three-Dimensional Structure of GSDMEs 

**Figure 6:**
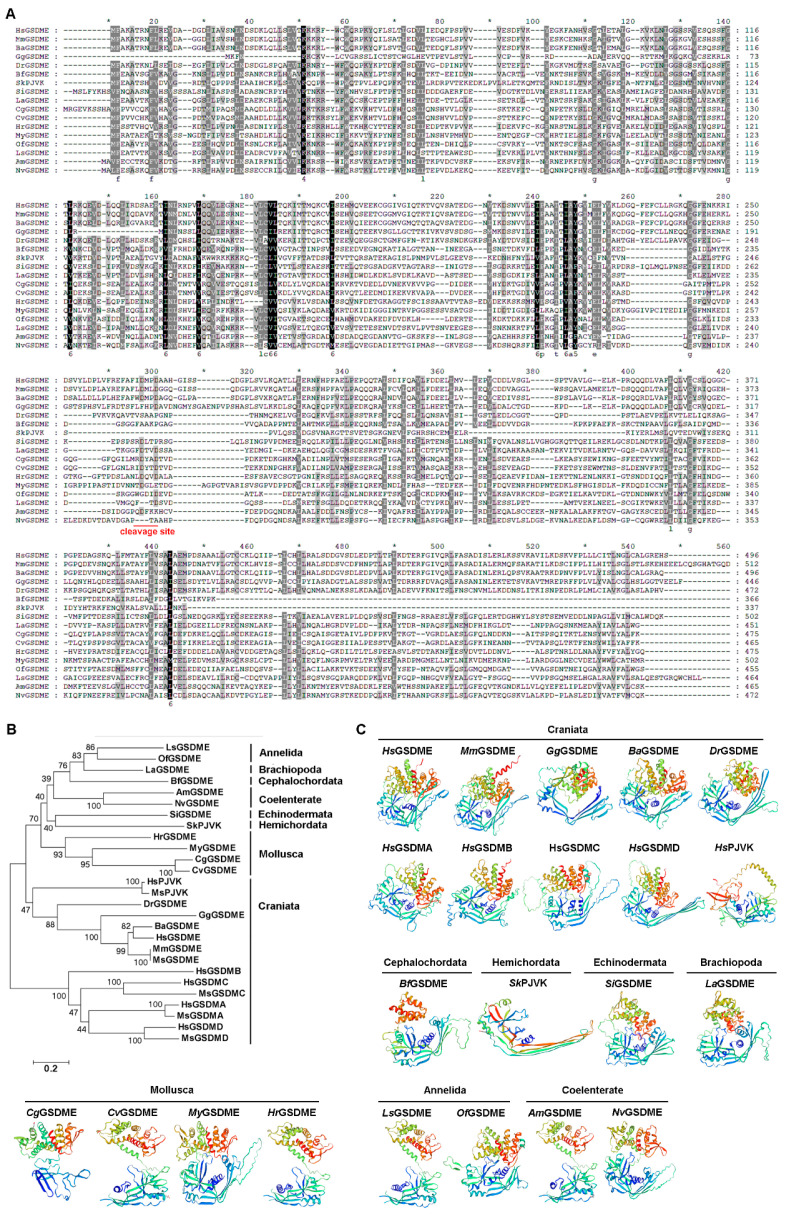
The multiple sequence alignment, evolutionary analysis, and three-dimensional structure of GSDMs from different species of metazoan phyla. (**A**) The multiple sequence alignment of GSDMs. (**B**) The evolutionary analysis of GSDMs. *A. millepora* XP_044165001.1; *N. vectensis* XP_001622420.1; *Owenia fusiformis* CAH1790265.1; *C. gigas* XP_034300415.1; *C. virginica* XP_022326088.1; *M. yessoensis* XP_021351819.1; *H. rufescens* XP_046378304.2; *L. anatina* XP_013387688.1; *L. satsuma* KAI0211901.1; *B. floridae* XP_035697721.1; *S. kowalevskii* XP_002740828.1; *S. intermedius* UYI58618.1; *D. rerio* NP_001001947.1; *G. gallus* XP_046767096.1; and *B. acutorostrata* XP_007171559.2. (**C**) The three-dimensional structure of GSDMs.

GSDMEs/PJVKs from species of different metazoan phyla were selected for analyzing the sequence similarity. They had lower sequence similarity, but their cleavage sites were relatively conserved (Figure 6A). According to the evolutionary tree, there were two clear branches of GSDMs in metazoa. GSDMA/B/C/D from human and mouse were dropped in one branch. All GSDMEs and PJVKs in metazoa were dropped into another branch, indicating that GSDMEs and PJVKs from human and mouse had closer evolution with those from lower vertebrates and invertebrates. In invertebrates, GSDMEs from the same animal phylum were clustered together and GSDMEs from Mollusca had relatively higher similarity with those from Craniata (Figure 6B). According to the evolutionary tree and three-dimensional structure, there were GSDMEs in different invertebrate phyla except for Hemichordata. The three-dimensional structures of GSDMEs from species of different metazoan phyla were very similar (Figure 6C).

### 2.7. The NLR Inflammasome Composition and Its Mediated Pyroptosis in Different Metazoan Phyla

**Figure 7:**
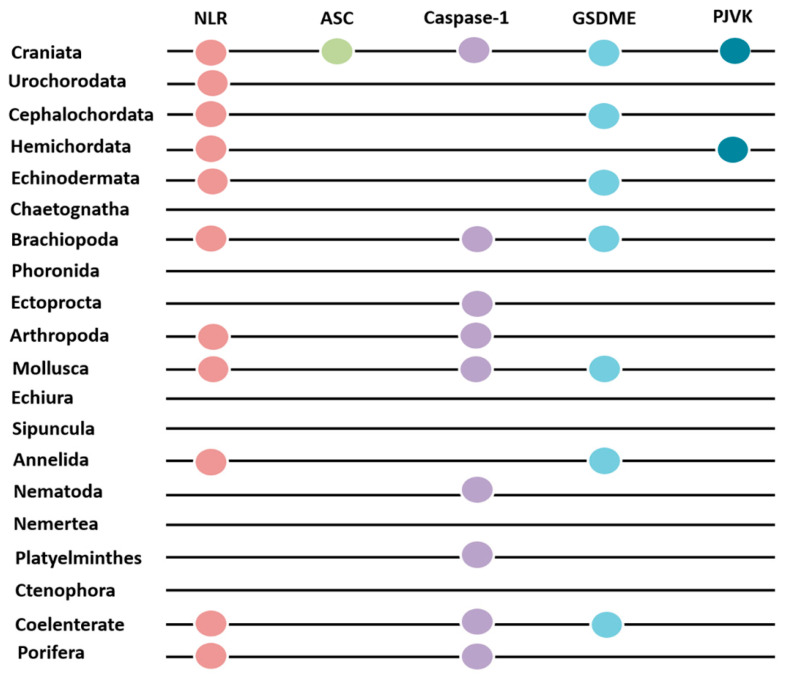
The existence of NLR, ASC, Caspase-1, and GSDM in different metazoan phyla.

NLRs, ASCs, Caspase-1s, GSDMEs, and PJVKs all existed in Craniata. There was only NLR existing in Urochorodata. NLR and GSDME/PJVK both existed in Cephalochordata, Hemichordata, and Echinodermata (Figure 7). NLR, Caspase-1, and GSDME all co-existed in Brachiopoda, Mollusca, Annelida, and Coelenterate. In Arthropoda and Porifera, there were only NLR and Caspase-1 (Figure 7).

**Figure 8:**
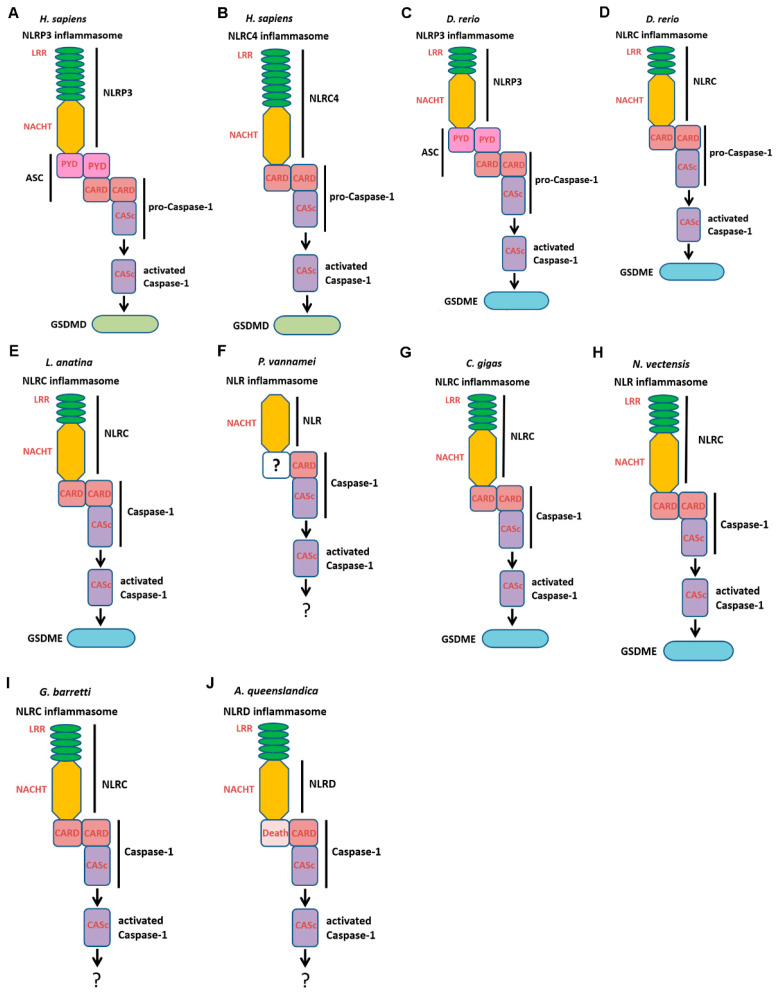
The schematic representation of NLR inflammasome and NLR inflammasome-dependent pyroptosis. (**A**–**D**) The NLRP and NLRC inflammasome and its dependent pyroptosis in *H. sapiens* and *D. rerio*. (**E**,**G**,**H**) The NLRC inflammasome and its dependent pyroptosis in *L. anatine*, *C. gigas*, and *N. vectensis*. (**F**,**I**,**J**) The NLRC inflammasome in *P. vannamei*, *G. barretti*, and *A. queenslandica*. “?” indicated there was no GSDM found in these species.

In *Homo sapiens* and *Danio rerio*, NLRP3 interacted with the PYD of ASC through its PYD and CARD of ASC then interacted with CARD of Caspase-1 (Figure 8A,C). In *H. sapiens*, the activated Caspase-1 bound to GSDMD to lead to the cleavage of GSDMD and in *D. rerio*, the activated Caspase-1 bound to GSDME to lead to the cleavage of GSDME (Figure 8A,C). NLRC4 from *H. sapiens* and NLRC from *D. rerio* directly interacted with CARD of Caspase-1 through its CARD (Figure 8B,D). The activated Caspase-1 then bound to human GSDMD or fish GSDME to lead to the cleavage of GSDMD/GSDME (Figure 8B,D). In *Lingula anatine* and *Crassostrea gigas*, NLRC interacted with CARD of Caspase-1 through its CARD (Figure 8E,G). The activated Caspase-1 then bound to GSDME to lead to the cleavage of GSDME. In *Penaeus vannamei*, NLR interacted with CARD of Caspase-1 through unknown domain (Figure 8F). In *Nematostella vectensis*, NLRC interacted with CARD of Caspase-1 through unknown domain (Figure 8H). And the activated Caspase-1 then bound to GSDME to lead to the cleavage of GSDME. In *Geodia barretti*, NLRC interacted with CARD of Caspase-1 through CARD to lead to the activation of Caspase-1 (Figure 8I). In *A. queenslandica*, NLRD interacted with CARD of Caspase-1 through Death domain to lead to the activation of Caspase-1 (Figure 8J).

## 3. Discussion

NLR inflammasomes are multiprotein signaling platforms that control the inflammatory response and coordinate antimicrobial defenses [1,3,26]. They are assembled following the detection of microbial signals and danger signals in the cytosol of host cells and activate Caspase-1 to lead to the maturation of pro-IL-1β and pro-IL-18 and induce pyroptosis [1,3]. Similarly to TLR4, NLRs represent a large family of multidomain proteins that were initially discovered for their role in host defense in vertebrates [27]. Over recent years, the wide distributions of NLRs and GSDMs among metazoa have become apparent [7,28,29]. In the present study, NLRs, Caspase-1s, and GSDMs presented from porifera to mammals were analyzed, the formation mechanism of NLR inflammasomes predicted, and their possible activation mechanisms and mediation of the process of pyroptosis were outlined. 

NLRs are a family of intracellular sentinels to be involved in both defense against microbes and cellular damage. Metazoan NLRs are defined by the presence of both a central NACHT domain and a series of C-terminal LRRs [3]. NLRs are classified into four distinct subfamilies in vertebrates: NLRA, NLRB, NLRC, and NLRP subfamilies [15]. NLRC and NLRP subfamilies have been characterized in some fishes, such as channel catfish, grass carp, and miiuy croaker [12,17,30]. Notably, NLR is absent in *Drosophila melanogaster* and *Caenorhabditis elegans* genomes. However, NLR gene families are significantly expanded in amphioxus, sea urchin, sea anemone, and hydra with respect to vertebrates [31,32,33,34]. In amphioxus and sea urchin, some NLRs use Death as the N-terminal domain, defined as NLRD [31]. CARD, PYD, Death, and Death effector domains are members of the death domain superfamily [35]. In the present study, NLRs from the genomes of species in different metazoan phyla were systematic screened and analyzed to investigate the evolution of NLRs in metazoa. In humans, there were four types of NLRs, NLRA, NLRB, NLRC, and NLRP. NLRC and NLRP also existed in fish. In invertebrates, there were NLRC and NLRD (a new type of NLR) presented. NLRD first appeared in sponge of metazoa, which was then presented in sea urchin and amphioxus, suggesting that NLRD of sea urchin and amphioxus might be evolved from sponge. There was no NLRD in vertebrates. The classical NLRC was also first presented in sponge, which then appeared in tubeworm, abalone, oyster, lingula, amphioxus, fish, and human, while NLRA, NLRB, and NLRP only existed in vertebrates. Therefore, it could be speculated that NLRC first appeared in the five types of NLRs and NLRD was only presented in some species of invertebrates. The N-terminus effector domain of NLRs is involved in protein/protein interaction and signal transmission while, in most other species, such as anemone, coral, shrimp, acorn worm, and ascidian, NLRCs all lacked the N-terminal domain, contained the NACHT-LRRs, or only NACHT domain, indicating that NLRC was not conserved in evolution and was associated with loss of LRRs and/or N-terminal domain, which also explained the domain variability of the vertebrate NLRs. Absences of NLR were observed across the invertebrate phyla (Phoronida, Ectoprocta, Echiura, Sipuncula, Nematoda, Nemertea, Platyelminthes, and Ctenophora). It is possible that in these species, NLR and NLR inflammasomes might have lost during evolution. According to the evolutionary tree, NLRs from different metazoan phyla (except for Cephalochorida, Echinoderma, and Coelenterata) were dropped in each separate branch among which, NLRs from Porifera were in the most primitive position and those from Craniata and Urochorodata were the highest in evolution. In the sub-branch of evolutionary tree, NLRs from Mollusca, Annelida, and Brachiopoda had relatively more recent evolutionary relationship with those from Craniata and Urochorodata. NLRs from Porifera were in the most primitive position and this identification of NLRC and NLRD in the genomes of sponges suggested that the ancestral NLRC and NLRD gene were already presented in the last common ancestor of metazoa.

NLRs exert their functions through interactions of the N-terminal effector domain with downstream adaptor proteins, effector kinases, and Caspases, often leading to inflammatory or apoptotic responses [1,36]. NLRPs interact with ASC through its PYD to activate pro-Caspase-1, and NLRCs can directly interact with the CARD of Caspase-1 through its CARD [5,36,37,38]. While, in invertebrates, the vertebrate ASC and even PYD are missing altogether. Therefore, the NLRP-ASC-Caspase-1 inflammasomes only existed in vertebrates. In metazoa except for Craniata, there only existed NLRC homologues or NLRD which only appeared in invertebrates. Moreover, it was speculated that in invertebrates, the NLR inflammasomes were composed of NLRC/NLRD-Caspase due to the absence of ASC. To confirm the existence of Caspase-1 homologues, the CARD-containing Caspases were all screened from different species’ genomes in invertebrates. In mammals, CARD-containing Caspases are Caspase-1, Caspase-2, Caspase-4, Caspase-5, Caspase-9, Caspase-11, and Caspase-12. According to the evolutionary tree, the Caspase-1 homologues existed in nine of nineteen phyla in invertebrates. NLRC inflammasomes lead to the formation of the catalytically active Caspase-1 [39]. In the present study, the sequence alignment of Caspase-1 homologues screened from the evolutionary tree of CARD-containing Caspases was conducted. And it was found that though the amino acid sequences of Caspase-1 homologues from different species of metazoa had a big difference; their activation sites (QACRG) were relatively conserved. The N-terminal domain of NLR is responsible for homotypic protein/protein interactions that initiate immune signaling pathways [26,36]. In Craniata, NLRC4 can interact with CARD of ASC or directly bind to Caspase-1 through its CARD [6,40]. In addition, the amino acid sequences of CARD/Death from NLRC and CARD from Caspase-1 homologue were highly conserved in *D. rerio*, *Branchiostoma belcheri*, *L. anatina*, *C. gigas*, *C. angulate*, *Mizuhopecten yessoensis*, *P. vannamei*, *Nematostella vectensis*, *A. queenslandica,* and *G. barretti*, respectively. Their structural domains were also very similar, suggesting that the NLRC/NLRD inflammasomes could interact with Caspase-1 homologue to lead to the activation of Caspase-1.

In mammals, pyroptosis can be triggered through both canonical and non-canonical inflammasome pathways [41]. Among which, the canonical inflammasome pathway is activated by NLR-Caspase-1, which can convert the pro-form of IL-1β and IL-18 into their mature, bioactive forms and induce pyroptosis. The IL-1β and IL-18 homologues were not found in invertebrates, but the GSDM homologues were identified [21,22,23,29,42]. In the present study, to systematically investigate the mechanism of inflammasome in mediating the pyroptosis, the possible GSDM homologues were screened from the genomes of different species in metazoa. GSDME were observed in Cephalochordata, Echinodermata, Brachiopoda, Mollusca, Annelida, and Coelenterate of invertebrate phyla, and PJVK was only in Hemichordata. Until now, there is still no report about the function of PJVK in pyroptosis. Therefore, it was speculated that GSDME-dependent pyroptosis was in Cephalochordata, Echinodermata, Brachiopoda, Mollusca, Annelida, and Coelenterate of invertebrate phyla. The earliest form of GSDME was in Coelenterate. Absences of GSDME were observed in Urochorodata, Chaetognatha, Phoronida, Ectoprocta, Arthropoda, Echiura, Sipuncula, Nematoda, and Nemertea of invertebrate phyla, suggesting that GSDM-mediated function might have been lost during evolution in these species. However, the amino acid sequences of GSDME/PJVK homologues from different species of metazoa had a big difference and their cleavage sites (DxxD) were relatively conserved. According to the evolutionary tree, there were two clear branches of GSDME/PJVK homologues from different species. GSDME/PJVK homologues from Craniata were in one branch and those from other animal phyla were in other branches. GSDME homologues from Mollusca had relatively more recent evolutionary relationship with those from Craniata. And the three-dimensional structures of GSDME homologues from different species were very similar, suggesting that they might all function in inducing the pyroptosis. 

At present, inflammasomes have been identified in vertebrates such as humans, mice, fish, etc. [12,26]. However, in invertebrates, although NLR homologues have been found, the existence of inflammasome and its formation mechanism remain unclear. In the present study, invertebrate species with potential inflammasomes were systematically analyzed as there was no PYD-containing ASC homologue identified in invertebrates and it was predicted that NLR could directly interact with Caspase-1 through its Death or CARD. NLR and Caspase-1 were found to co-exist in the same species of Brachiopoda, Ectoprocta, Arthropoda, Mollusca, Annelida, Coelenterate, and Porifera. However, NLR in Arthropoda lacked the N-terminal effector domain. Therefore, it was possible that the presence of inflammasome was in these phyla except for Arthropoda. The activation of inflammasomes leads to the activation the proteolytic cleavage of GSDMD to induce pyroptosis [36], while in invertebrates, there was only GSDME or PJVK found [29] and PJVK exhibited no pyroptosis-inducing capacity [43,44] suggesting that in invertebrates, the activation of NLR inflammasomes could activate the proteolytic cleavage of GSDME to induce pyroptosis. In the present study, it was predicted that the NLR inflammasome-induced cleavage of GSDME existed in Brachiopoda, Mollusca, Annelida and Coelenterate, suggesting that the function of inflammasome in inducing pyroptosis evolved from Coelenterate. Also, in vertebrates, the activation of inflammasome induces the cleavage of IL-1β and IL-18, while in invertebrates, there are still no IL-1β and IL-18 homologues found in the genomes of all species, indicating that the function of inflammasome in inducing the cleavage of IL-1β and IL-18 was unique in vertebrates. In invertebrates, though there were no IL-1β and IL-18 homologues, there were other inflammatory cytokines [45], suggesting that in these species, the activation of inflammasome might mediate the activation and releases of these inflammatory cytokines.

In conclusion, NLR, Caspase-1, and GSDME emerged early in invertebrates of metazoa and had undergone dynamic evolution. NLRC, NLRD, and Caspase-1 as well as NLR inflammasomes evolved early from Porifera of metazoa. And their sequence and structural domains had changed greatly in evolution leading to the absences of NLR inflammasome in most metazoan phyla. NLR inflammasome, GSDME, and pyroptosis first co-existed in Coelenterate of metazoa, suggesting that the function of NLR inflammasome in pyroptosis evolved from Coelenterate. And this function was not conserved in evolution, and they only existed in some metazoan phyla, such as Annelida, Mollusca, and Brachiopoda. Though the activation mechanism of NLR-Caspase-1-GSDME was predicted in Annelida, Mollusca, and Brachiopoda, more experimental evidence is required to confirm the hypothesis. Together, these results shed new light on the composition, origin, evolution, and function of NLR inflammasome.

## 4. Materials and Methods

### 4.1. The Amino Acid Sequences and BLASTp Analysis of NLRs, ASCs, CARD-Containing Caspases, and GSDMs

The amino acid sequences of NLRs, ASCs, CARD-Containing Caspases, and GSDMs were obtained by screening the genomes of different species in metazoan subphyla/phyla from the National Center for Biotechnology Information (NCBI) database (https://www.ncbi.nlm.nih.gov/, accessed on 5 April 2024). The BLASTp were used to search the homologues of NLRs, ASCs, CARD-Containing Caspases, and GSDMs in the genomes of different species.

### 4.2. The Evolutionary Analysis of NLRs, CARD-Containing Caspases, and GSDMs

The phylogenetic trees of NLRs, CARD-Containing Caspases, and GSDMs were constructed with MEGA 7 software with the neighbor-joining (NJ) method, respectively. The bootstrap of 1000 replications was conducted to evaluate the phylogenetic tree.

### 4.3. Multiple Sequence Alignment of NLRs, ASCs, Caspase-1s, and GSDMs

The multiple sequence alignment of ASCs, Caspase-1s, and GSDMEs from different species of metazoan phyla was generated via MEGA 7 and GeneDoc-32-v320 software. The multiple sequence alignment of PYD/CARD/Death domain in NLRs and Caspase-1s from the same species was also generated via MEGA 7 and GeneDoc-32-v320 software.

### 4.4. The Structural Domain and Three-Dimensional Structure Analysis 

The structural domains of NLRs, ASCs, Caspase-1s, and GSDMs from the major metazoan phyla were predicted via the Simple Modular Architecture Research Tool (SMART) (http://smart.embl.de/ accessed on 5 April 2024). The three-dimensional structure of NLRs, ASCs, Caspase-1s, and GSDMs were predicted by using SWISS-MODEL (https://swissmodel.expasy.org/interactive, accessed on 5 April 2024).

## Data Availability

Data is contained within the article and Supplementary Materials.

## Supplementary Materials

The following supporting information can be downloaded at: https://www.mdpi.com/article/10.3390/ijms252011167/s1.
