# Supplementary material for: The Evolution of NLR Inflammasome and Its Mediated Pyroptosis in Metazoa
Source: Int J Mol Sci. 2024 Oct 17;25(20):11167. doi: 10.3390/ijms252011167 (PMC11508797; doi:10.3390/ijms252011167)
Supplement: Supplementary file 1 [file ijms-25-11167-s001.zip › ijms-3270209-supplementary.pdf]

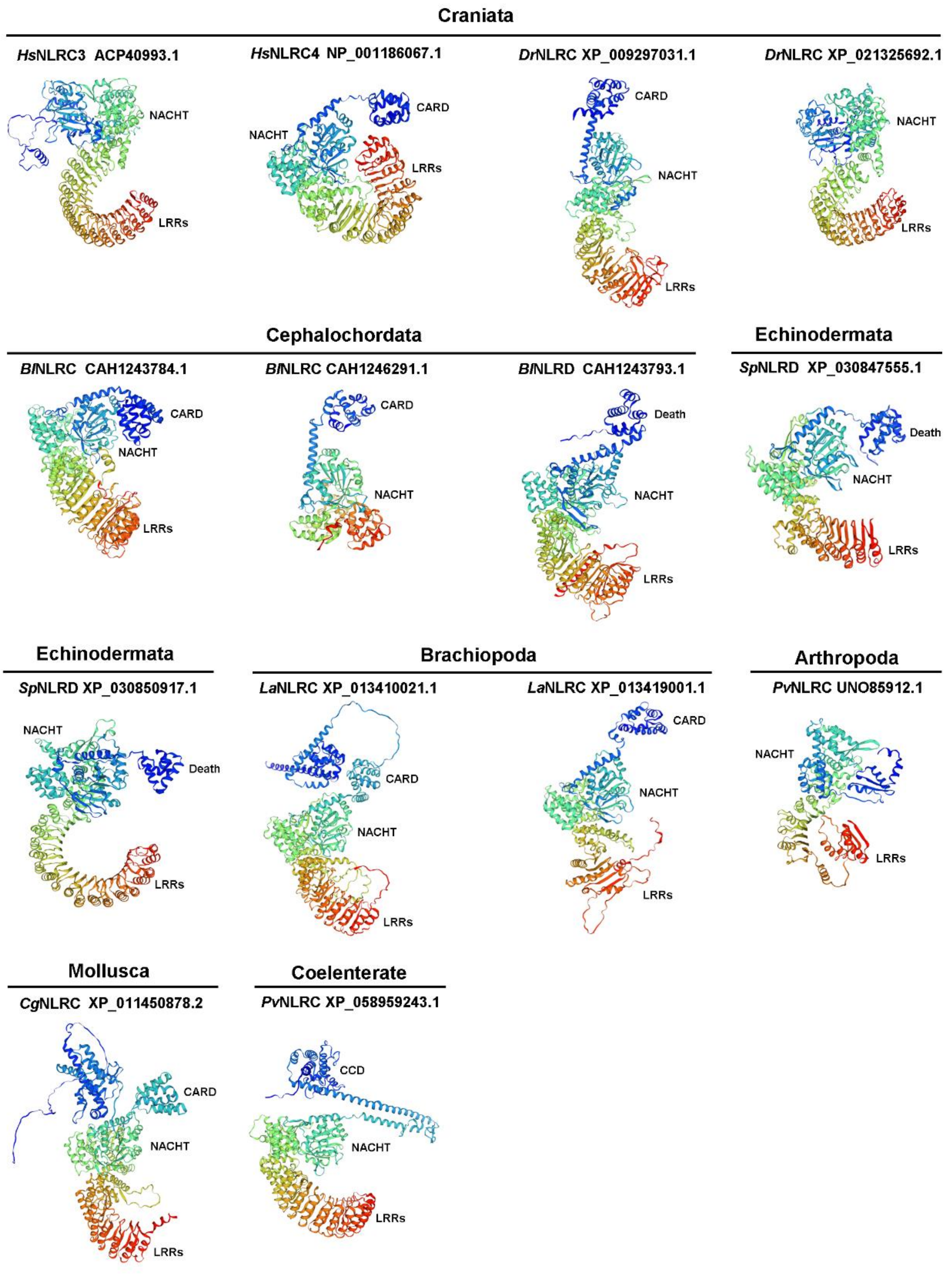

Figure S1. The three-dimensional structure of NLRs in different metazoan phyla.

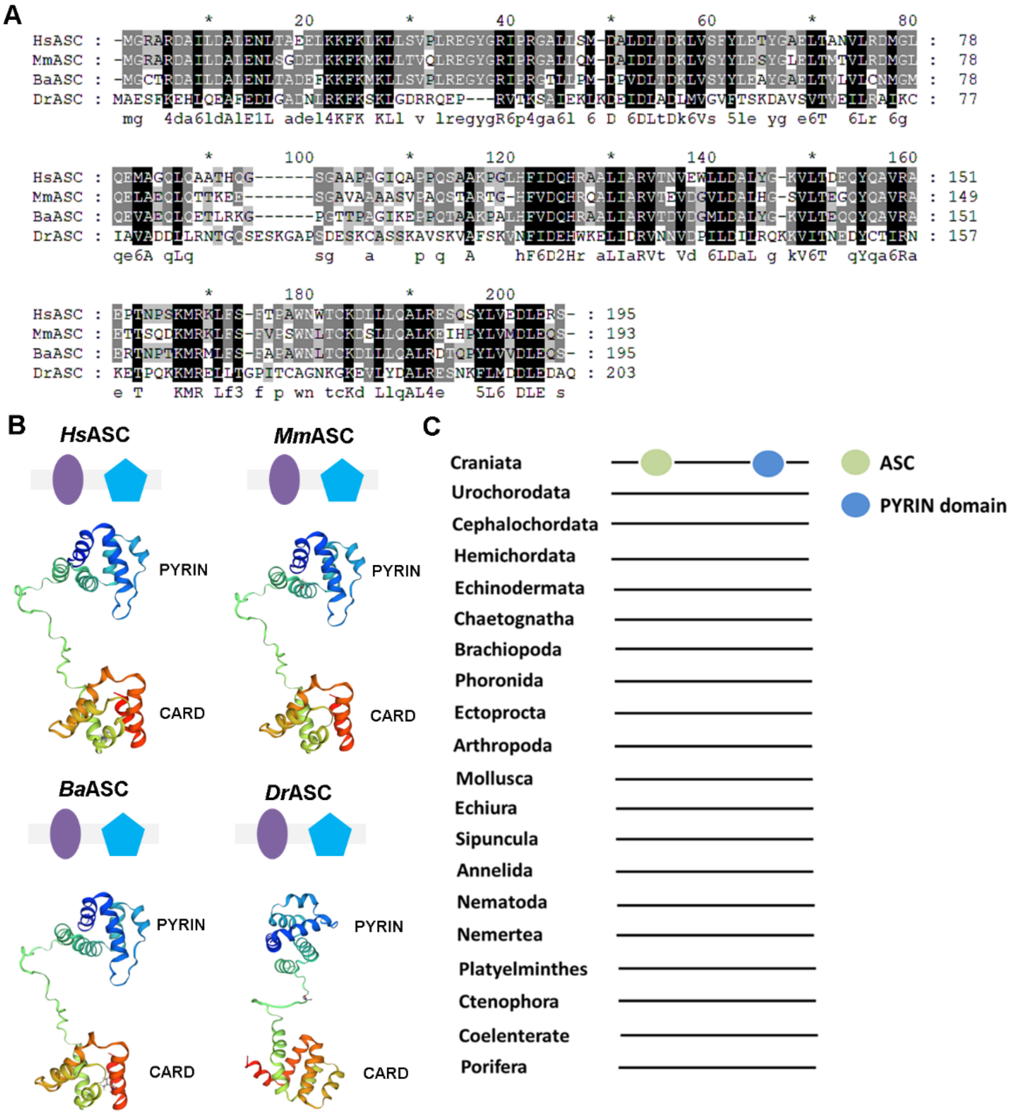

Figure S2. The multiple sequence alignment, three-dimensional structure and existence of ASCs in metazoan phyla. A. multiple sequence alignment of ASCs in vertebrates. *H. sapiens* ASC, NP_037390.2; *M. musculus* ASC, BAB97214.1; *B. acutorostrata* ASC, XP_007186519.2; *D. rerio* ASC, AAH95025.1. B. The three-dimensional structure and structural domains of ASCs in vertebrates. C. The existence of ASCs and PYRIN domain in different metazoan phyla.

Table S1. The abbreviation used in this study.

Abbreviation	Full name	Abbreviation	Full name
NLR	nucleotide-binding oligomerization domain (NOD)-like receptor	CARD	caspase recruitment domain
ASC	apoptosis-associated speck-like protein containing a caspase recruitment domain	pro-IL-1β	pro-Interleukin 1β
pro-IL-18	pro-Interleukin-18	PYD	pyrin domain
NACHT	nucleotide-binding oligomerization	LRR	leucine-rich repeat
GSDM	gasdermin	PJVK	Pejvakin
BIR	baculoviral IAP repeat	FIIND	function to find domain
PRY-SPRY	B30.2	TLR4	Toll-like receptor 4
GT	glycosyltransferase	AD	acidic transactivation
NCBI	National Center for Biotechnology Information	NJ	neighbor-joining
SMART	Simple Modular Architecture Research Tool	*Geodia barretti*	*G. barretti*
Amphimedon queenslandica	*A. queenslandica*	*Acropora millepora*	*A. millepora*
Nematostella vectensis	*N. vectensis*	*Schistosoma japonicum*	*S. japonicum*
Clonorchis sinensis	*C. sinensis*	*Caenorhabditis elegans*	*C. elegans*
Lamellibrachia satsuma	*L. satsuma*	*Crassostrea gigas*	*C. gigas*
Crassostrea angulata	*C. angulata*	*Mizuhopecten yessoensis*	*M. yessoensis*
Haliotis rufescens	*H. rufescens*	*Aplysia californica*	*A. californica*
Lingula anatina	*L. anatina*	*Saccoglossus kowalevskii*	*S. kowalevskii*
Apostichopus japonicus	*A. japonicus*	*Strongylocentrotus purpuratus*	*S. purpuratus*
Penaeus vannamei	*P. vannamei*	*Penaeus japonicus*	*P. japonicus*
Branchiostoma belcheri	*B. belcheri*	*Ciona intestinalis*	*C. intestinalis*
Danio rerio	*D. rerio*	*Homo sapiens*	*H. sapiens*
Mus musculus	*M. musculus*	*Gallus gallus*	*G. gallus*
Balaenoptera acutorostrata	*B. acutorostrata*		
